# Change and Aging Senescence as an Adaptation

**DOI:** 10.1371/journal.pone.0024328

**Published:** 2011-09-16

**Authors:** André C. R. Martins

**Affiliations:** 1 GRIFE – Escola de Artes, Ciências e Humanidades, Universidade de São Paulo, São Paulo, Brazil; 2 Centre de Recherche et Epistemologie Appliquée, École Polytechnique, Palaiseau, France; University of Zürich, Switzerland

## Abstract

Understanding why we age is a long-lived open problem in evolutionary biology. Aging is prejudicial to the individual, and evolutionary forces should prevent it, but many species show signs of senescence as individuals age. Here, I will propose a model for aging based on assumptions that are compatible with evolutionary theory: i) competition is between individuals; ii) there is some degree of locality, so quite often competition will be between parents and their progeny; iii) optimal conditions are not stationary, and mutation helps each species to keep competitive. When conditions change, a senescent species can drive immortal competitors to extinction. This counter-intuitive result arises from the pruning caused by the death of elder individuals. When there is change and mutation, each generation is slightly better adapted to the new conditions, but some older individuals survive by chance. Senescence can eliminate those from the genetic pool. Even though individual selection forces can sometimes win over group selection ones, it is not exactly the individual that is selected but its lineage. While senescence damages the individuals and has an evolutionary cost, it has a benefit of its own. It allows each lineage to adapt faster to changing conditions. We age because the world changes.

## Introduction

Living organisms shouldn't age, at least if that could be helped. They show a remarkable capacity of repairing different kinds of damage and there is no physical reason why damage caused by simple passage of time couldn't also be repaired. Evolution works in a way that any species whose representatives have any distinct disadvantage will be driven to extinction. It makes sense then to assume that, if aging could be avoided, species that showed senescence as the individuals grow older should be replaced by others where aging does not happen (or happens at a much slower rate). Senescence increases mortality and an individual who dies of old age will leave, on average, a smaller number of descendants than another individual that does not age and manages to live and reproduce for a longer time. And yet many known living organisms show senescence. The time it takes for an individual to show signs of old age varies greatly among species, but aging seems so natural that many people fail to realize there is an apparent contradiction between senescence and evolution. Understanding the reasons why animals do age, despite or because of evolution, has important consequences in the prospect of whether aging is something we can avoid or not. If there is a genetic program for getting older, researchers can try to find ways to turn that mechanism off, with profound medical and demographical implications [1], [2]. But such a program would not be observed if it provided no evolutionary advantage.

General theories of aging are too many to be all listed here [3]. Many attempt to describe the mechanisms behind aging. However, while understanding the exact biological processes that lead to aging is fundamental, especially if we want to be able to counter its effects, it does leave one important question unanswered. That is the question of why. Since aging is so pervasive, even though there are many organisms that shows no signs of senescence [4], [5], it seems reasonable to conclude that there might be general common principles behind it. The natural question becomes, from an evolutionary point of view, why most organisms have evolved to a situation where the individuals are, in some sense, harmed by some senescence mechanism [6]. We should notice that the usual claim that there is irreparable damage caused by time itself is in contradiction with data. Not only the age of senescence of organisms with similar characteristics, including similar biochemistry varies wildly, but, if senescence was a basic law of nature, there would be no species where it wouldn't be observed. Wear and tear theories might be useful to explain how we age; they don't provide the answer to the question of why we do.

At first, given the absence of many elder individuals in natural conditions, it was believed that any detrimental effects will have lesser evolutionary importance if those effects only happen at advanced ages, since fewer individuals would be subject to it [7]. If, as generations went by, detrimental mutations happened that caused damage to the individuals only during older ages, evolution would be too weak to cause the extinction of those mutations. However, more recent observations have concluded that, for several species, senescence is actually a cause of many deaths [8]. And, therefore, it is not something that would have no evolutionary importance or effect.

This has caused the appearance of theories that try to explain how senescence can exist in context of evolution and adaptation. Antagonistic pleiotropy theory [9], per example, proposes that there might be genetic adaptations that, while providing benefits early in life, could be associated with problems later on. Since many more animals would receive the benefits, as the rate of survival to old age is small, those benefits can compensate the posterior damage. Conditions for optimality for the theory have been studied [10], showing that, if some genes fit the description of the antagonistic pleiotropy hypothesis, there are circumstances where evolution will work in favor of keeping those genes. This does not mean, however, that most of the aging process is based on this effect. Long term laboratory experiments have shown that drosophilas can be altered through selection so that their life span will be longer without any harmful effects in fertility, clearly contradicting pleiotropic theories [11]. Their aging is not caused by some early beneficial effect.

Another evolutionary theory of aging that is based on individual selection is the disposable soma theory [12]–[15]. The idea is that all animals need to have repair and maintenance systems and use limited food energy to keep them functioning properly. These systems need to work during most of the reproductive life of an animal. However, since most animals in the wild die relatively young [16], there would be no need for assigning valuable resources and energy to a perfect repair system that could keep the animals functioning longer. Optimal levels of maintenance would be dependent on the environment and species, thus explaining why different species age at very different rates. Evidence that species that are less subject to early accidental deaths live longer do exist and analysis of the life spans of social insects do seem to agree with the qualitative predictions of the theory [17].

These theories, while possibly correct for several specific cases, are not capable, however, of explaining the whole range of observations related to aging. Accumulated mutation theory predicts that all organisms should show signs of senescence and that those organisms should be increasingly damaged the longer they live. However, not only some animals show no signs of aging, some, like female turtles [18], show evidence of increasing fertility with aging, in the contrary direction to what the theory predicts. Disposable some theory predicts that, if some mutation would increase the life span of an animal, it should decrease its metabolism, since resources would have to be diverted from other functions. However, genetic mutations that allow for longer life and cause no other losses have been observed [19]–[21]. Molecular genetic studies have also shown that longevity can be subject to regulation and interventions can even reverse aging in animals [22]. At this point, it seems that aging might not be completely connected to fertility. It is possible for animals to live longer without costs to their early life fertility. There is now a fast growing body of evidence that points to the conclusion that aging is a genetic mechanism that evolution could have not chosen to use [23], [24]. This makes an evolutionary explanation for why we age a very strong theoretical need.

We are led to conclude that senescence might actually be an adaptation by itself, that it might not be a detrimental consequence of other gains. If that were the case, it would have to offer benefits. That is, despite the apparent contradiction that harming individuals shouldn't be a good evolutionary solution, senescence would actually be helping those individuals to somehow increase the number of their descendants. Since this is an apparent contradiction, explanations and new models are needed to explain the gap between theory and data. A natural candidate to why individual selection could actually lead to an adaptation that can harm the individual interest is the concept of kin selection [25]–[27]. It might be in the best interest of the parents (in the sense of increasing the expected number of descendants) to die of senescence, leaving the available resources to their offspring [28]. Spatial models [29], [30] have also been proposed but their results might be a consequence of kin selection effects. The introduction of diseases in a spatial model was also shown to have a positive influence into the adoption of senescence [31]. And a model that ties senescence with the fact that organisms grow was also also proposed [32].

Kin selection certainly plays an important role in the evolution of senescence. However, there is one part of this solution that sounds like circular reasoning. In cooperation problems, kin selection is actually a very good strategy because adult offspring is expected to produce larger progenies than their parents. This is particularly true when animals age. Parents are older, therefore, if senescence exists, they have an expected average number of children smaller than the number of their children. By transferring the resources to their offspring, they actually increase the expected number of their descendants. This reasoning fails when applied to senescence. If animals didn't age, there is no reason to assume that the children will be around longer so that they can produce more offspring than their parents. The expected gain will not be important unless there are mechanisms that give an investment in the offspring a better expected return than keeping the resources for the parent. It might even be non-existent. Also, there are several results in the literature about cooperation pointing to the fact that while it makes sense for individuals to be altruistic to their relatives, if competition happens among those same relatives, the beneficial effects of kin selection can become smaller or disappear [33], [34]. While there are conditions where kin selection has an impact on the competition [35], [36], we will see evidence on the simulations that this is not the only thing happening in the models presented here. Kin selection (and, therefore, group selection) does play a role, through viscosity, but it alone does not explain aging, as we will see.

I will show that this new conundrum can be solved if one notices a characteristic of the recent models, as they have some characteristics in common. In spatial models, conditions are different in each site and change over time. Diseases come and go, also introducing some non-stationarity characteristics into the problem. In the real world, change is actually much more common than in most evolutionary models. The environment changes, other species evolve, mutations happen. The conditions our ancestors were well adapted to are not necessarily the same ones as the conditions we have to live in.

In this paper, I will present a model where two initially identical species compete for supremacy in a spatial grid. The only difference between them, at first, is that one of them dies of senescence, while the organisms of the second species could, in principle, live forever, if no accidents or competition happened. Each individual will be characterized by its ability to survive one more time step and, at each time step, alive organisms can produce offspring. In order to reflect the changing in the environment without actually having to worry about specifics, the fitness function, representing the survival capability of an individual, will decrease at each time step by a small amount. Also, offspring will not be an exact copy of their parents and, as such, their fitness can the the same, worse or better. I will show that when there is change, survival of an aging species can be the chosen by individual competition, provided there is viscosity to allow for some amount of group selection.

The mechanics of the interaction between specific individuals in the model presented here includes only individual competition. They fight for survival and the one with higher fitness will have a higher probability to win the competition. No direct use of relatedness or group are used in the choice of who survives. However, the agents are placed in a spatial structure, represented by a grid and generations move slowly on that grid. That is, there is population viscosity and, as such, the observed effect will be due also to group selection [37]. In this sense, kin selection is part of what happens and a partial cause to it (and so, also group selection, since it is formally equivalent to kin selection [38]).

## Methods

### A Model for Change

In the model presented here, individuals will compete in a landscape representing the world where they live. The environment will be represented by a square two-dimensional grid with 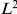 sites, so that each individual will live and compete for the resources in one of the sites. Periodic boundary conditions will be assumed, so that no boundary effects are observed. Each site will have a carrying capacity 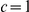, that is, it can only sustain one individual at a time. Whenever more than 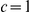 individuals share the same site, they will compete for the local resources and only one will survive. Time will be measured in discrete steps, each time step corresponding to one generation, that is, the time the organisms need to produce new viable offspring. New offspring does not represent all the children of one individual, since only individuals who are at reproductive age are modeled. As such, if one species has many children and most of them die before reaching maturity, only the surviving child is described in the model and all others are assumed to have died between time steps.

While most traditional evolutionary models work with a stationary environment as basis, which can be a very good first approximation, real world conditions are not unchanging. Climatic cycles happen, predators and prey evolve together in constant evolution, new diseases appear and replace old ones. Trying to model all those aspects and how they change with time would be a daunting task, with too many yet unsolved questions, and that would also unnecessarily complicate the model. Instead of doing that, an approximation will be adopted here. This will be implemented by proposing a type of fitness function, that captures the influence of the environment and the changing conditions. Unlike fitness functions of Evolutionary Game Theory [39]–[41], the one we will use here is not exactly the final payoff of a game the individuals play. But it plays a similar role, as it is related to the likelihood an individual will survive sharing resources with a competitor.

Let 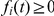 be a fitness function, such that whenever there is competition between two (or more) agents in one site, the probability that agent 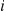 will prevail is proportional to 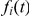. Given agent 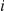 and agent 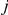 competing for the resources in a site at time 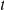, agent 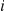 will survive with probability 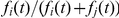. The larger 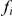 is, the more likely 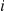 is to survive, but there will always be a chance that less fit individuals would survive (except, of course, when 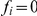).

At each time step, surviving individuals produce offspring. Each offspring is born at a distance 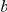 from its parent, where 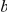 is measured in units of the grid size and it inherits the fitness of its parents, except for small deviations, due to mutation. For simplicity, if organism 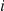 is the parent of organism 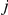, the fitness of 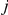 will be 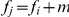, where 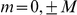, with equal probabilities for the three possible results. This represents small changes. Cases where rare, large and usually detrimental mutations happen will not be included in the model here, as strong detrimental mutations would almost certainly not survive until adulthood. The mutation here only represents the fact that surviving offspring can be a little different from their parents . Also, in order to represent the fact that conditions change, 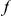 is diminished by a constant value every time step, so that 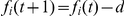, where 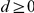 for every individual 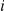.

Some comments about the meaning of 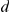 are necessary here. As described above, 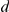 might represent a rate of change in the environment. However, it also plays the mathematical role of keeping the fitness from exploding due to mutation. We will see that 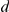 and 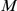 are related in a way that makes it very hard to define 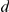 as a simple measurement of environment change. Environment change can have an impact on 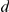 but an exact interpretation of its values can be misleading due to its connection with the mutation.

Two types of animals are introduced, those who die of senescence and those who will only die due to other competition. At first, both types will always start with the same values for their parameters. In the model, all organisms who suffer the effects of senescence and will die at the same programmed age, 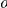. It should be noted that, for very small values of 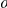, aging is clearly a very important disadvantage as many individuals will die too fast. On the other hand, very large values of 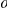 have a very small effect, because almost no individuals would survive long enough to reach a very large 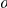. Tests for 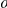 as large as 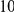 show basically equal chances for both species, since so few individuals survive that much, and any aging effects become almost negligible. Therefore, we will limit the simulations to values of 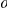 between 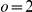 and 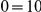 in the following Sections. In order to introduce the possibility of random deaths, disassociated from the competition, a chance 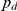 that each organism will die at each time step could also be easily introduced. Preliminary tests with different values of 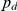 showed little difference in the final chances of extinction for each species. Therefore, this will not be further explored in this paper.

## Results

### Aging and Competition

It is, of course, fundamental that the model, when no changes happen, should reproduce some basic results of evolutionary aging theory. First, when the system is completely stable, no mutation going on and no changing conditions for worse, that is 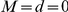, its is to be expected that a population that shows senescence will be driven to extinction. This happens simply because its members will die faster. And this is indeed the case. As a test, 20 runs were performed on a 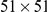 bi-dimensional grid, using NetLogo as platform [42], where the species with senescence just survived until 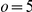 time steps. Offspring was born at a distance 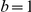. The species with senescence always became extinct after 220–230 time steps. While this is not immediate, each time step corresponding to a new generation being born, the initial decline was still fast and the end, unavoidable.

That happens despite the fact that competition did cause old animals to be rare, both between the senescent and non-senescent species, with only approximately half of the individuals surviving longer than one time step. Despite the small numbers of elder individuals, the cost is enough to prevent any possibility of survival for the aging species. Different values of the dispersal distance of new births, 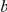, provide the same scenario, with just different time scales for the extinction of the senescent species to happen. Per example, if diffusion is really slow, with 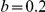, it takes about 1,800 generations, in average, for the aging species to die out.

### Mutation

More interesting effects appear when change is introduced. Even with a fixed environment and change brought only as mutation, we can already observe new effects. The mutation introduces a random element that affects the competition between the species, with the obvious possibility that one of the species will get a better fitness 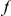 by simple random chance. This should have a similar impact on both species and, at first sight, should not cause any qualitative differences from the results where no mutation happened.

However, what we see is that there is a clear tendency for the aging species to adapt faster, meaning that 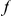 has a strong tendency to be larger for the species that experiences senescence. This adaptability somehow compensates part of the detrimental effect that death by senescence has in the species. In a number of runs, it was observed that, for a while, the tendency to extinction of the senescent species was even reversed and its population increased in size, when the difference between average values of 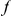 was large enough. Even though senescence still caused the extinction of the aging species in most runs, it took longer for extinction to happen. And, even more important, there were a few cases where the aging group actually led the non-aging group to extinction!

As we increase the effects of mutation by making 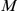 larger, per example, 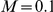 (and born distance 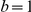), we see that the non-senescent species finds it more difficult to drive the senescent one to extinction. While we observed extinction after an average of a little more than 200 generations when no mutation happened, the new average time to extinction grows to about 1,000 generations. Also, instead of a simple massacre where the non-aging species wins in every run, we observed that 7 out of 50 runs ended with the non-aging group extinct.

Notice that this does not seem to be only a kin selection effect, as the descendants of the non-aging group are exactly as important to the survival of the species as their parents. The benefits of the senescence will be most likely used by the descendants or relatives of the individual who died of senescence and, therefore, the results have a strong influence of group or kin selection. However, it is not always true that a relative will be the beneficiary of the death. The chance that a close relative will benefit obviously decreases with the distance 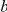. For larger 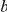, diffusion is faster and descendants tend to live further from their parents. Per example, when 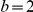, and it is guaranteed that the parents will never compete with their direct offspring. However, 5 out of 50 runs ended with the aging group as the only survivors. An even stronger evidence against the idea the kin selection might be the only responsible for the agers survival is observed when 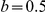. Such a small displacement means that quite often parents will compete with their offspring, however, there was not a single run out of 50 where the aging species won at the end. Kin selection effects ought to be stronger the more the parents would compete with their offspring. There is also something else going on here, as an effect of the non-stationarity and mutation.

The eventual victory of the aging species in those few cases is almost certainly due to size effects and random fluctuation. And, indeed, when running a larger system with 101×101 sites, no victory of the aging species was observed in 20 realizations. However, the fact that finite size effects become more important shows that mutation makes aging more competitive. The increase in the competition brought by mutation is an important force in the system. And, in this case, the faster increase in the fitness function for aging species can actually make an important difference.

### Environmental changes

Introducing the idea that fitness decreases with time takes the model one more step closer to a more correct description of changes in the real world. As described above, this can be implemented by decreasing all 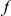
s by a constant amount, 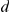, at each time step (generation). It is obviously an approximation to consider that the changes will affect all individuals equally and more random effects should be studied in the future. With the change in the environment, some degree of mutation is crucial, otherwise all 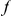
s will decline to zero fast. Also, if the mutation were too weak, selection forces wouldn't be able to keep up with the change in the environment. While this has minor consequences (up to the point where 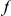 would become zero) in the model, as only the two species compete for the resources, in the real world, with more competitors, that could mean extinction for everyone.

It is interesting to notice that, since individuals survive a conflict according to a probability given by the ratio of their fitness, instead of their exact values, the function 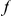 can be arbitrarily multiplied by any constant with absolutely no changes in the model, as long as the same constant multiplies the fitness of every agent. What we observe in every realization is that, regardless of the initial fitness, as the system evolves, it will reach a state where the fitness of each group oscillates around a value that depends strongly on both the non-stationary decrease 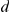 as well as the mutation rate, 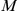 (except for the cases when 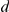 is too small and the average fitness explodes). The reason the average fitness can start changing and then stabilizes around some value is easy to understand. Basically, 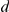 causes the fitness to diminish and 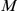 introduces a competition where larger fitness tend to be more important. If average fitness of the agents is much larger than 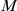, the advantage it gives is too weak and 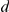 dominates, making average fitness decrease. However, the system will eventually reach a point where 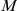 will provide an important advantage and, unless 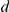 is too large, this will cause the fitness to stabilize around some value. In this sense, despite it is fundamental that both parameters exist, only one is really independent. The tests we will conduct will be perfiormed varying only the value of 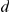.

#### Understanding the model dynamics

Before exploring the whole space of parameters, it is useful to understand the dynamics of the model better. To achieve this goal, few cases will be explored here in this Subsection. The choice of the cases at this point is based on which cases are good examples of how the system evolves. After these examples, in the next Subsection, a more complete exploration of the parameter space will be presented. There, we will see how likely agers are to cause the extinction of non-agers for several different values of the parameters.

The first thing we observe by introducing a decrease in the fitness associated with mutation is that the dynamics is altered in a way that makes ager survival much easier. Per example, introducing a tendency for the fitness to decrease (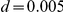) with time in the case described in the end of Subsection 0 (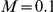 and 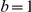) led to just a small increase in the number of victories by the aging group (10 in 50, instead of 7 in 50). For, 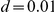, it is no longer clear, for just a few simulations in a finite lattice, which group has the advantage (23 ager victories in 50). For a larger system, with a grid of size 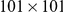 (effectively allowing for four times the number of individuals in the system), the number of victories of the aging species actually increased to 39 in 50.

**Figure 1:**
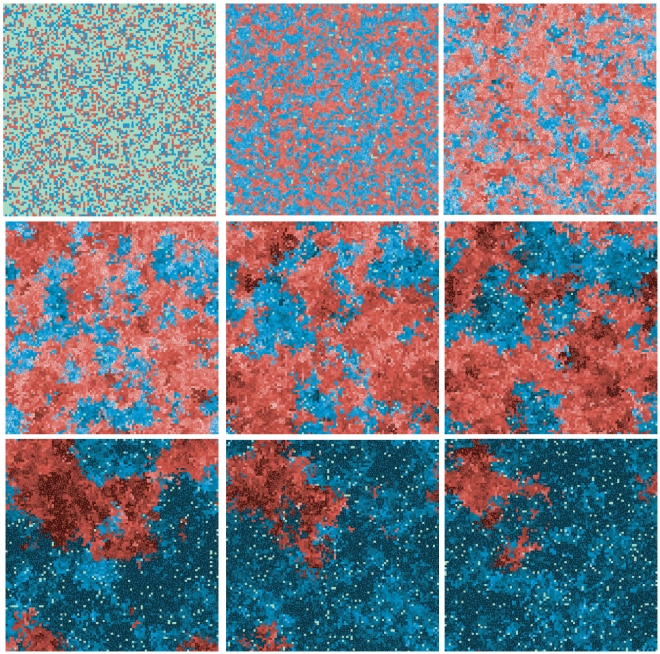
The spatial evolution of a typical run. Blue squares correspond to a presence of an ager agent and red to the presence of a non-ager in that location. The figures show the same realization of the problem at different times, from the beginning (top left), evolving from left to right and then from top to bottom. Both for blue and red, lighter tones indicate a smaller fitness, while darker tones correspond to a higher fitness. Green spots correspond to places where no living agent is present.

It is also interesting to investigate how the system evolves with time. Figure 1 shows how each group disperesl and the evolution in time of spatial configuration in a typical run of a 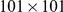 grid, with parameters 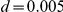, 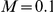, and 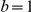. Blue squares signal the presence of an ager agent and red, of a non-ager in that location. Green spots correspond to places where no living agent is present. In the first instant (top, left), the system is initialized with a fixed number of agents, equal for both groups, each located by chance. Therefore, many places still have no agents in them and that is the reason for the larger amount of green there. Soon after, the whole lattice is occupied and green appears only when an ager die of senescence and no offspring has occupied the spot yet. The fitness of each agent is represented by the tone of the color, a higher fitness showing as darker tones. We see that, initially, the distribution is basically random. As time goes by, each small local groups disappear as some die and others grow to much larger sizes. While non-agers seem to have an initial advantage, as fitness start to change, the agers are finally able, in this realization, to start driving non-agers to extinction.

**Figure 2:**
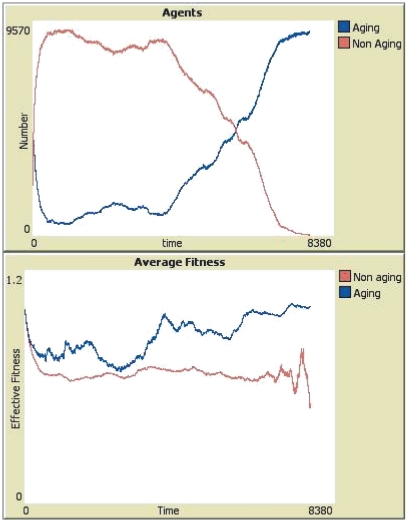
The evolution of a typical run that ended with the victory of the senescent species. The blue lines correspond to the species where there is senescence (aging) and the red lines to the species where individuals do not age. *Top Panel*: Number of individuals (agents) in each group as a function of the number of generations (time). *Bottom Panel*: Evolution of the average value of the fitness function for each species.

This happens because the average fitness of the agers is actually larger than the average fitness of non-agers. A typical evolution of the average fitness in a run ending with a victory of the aging species can be seen in Figure 2. It is easy to notice that the system is initially in a transient phase, due to non-natural initial conditions. When the simulation starts, an equal number of individuals of each species is created. Their location is randomly assigned and every agent has the same age of zero. As all spots are occupied soon after the start of the run, competition is unavoidable. The groups coalesce, with each species surviving in different areas, both as an effect of the competition and of chance. As expected, as the aging species starts losing a few of its members to senescence, the total number of aging individuals start to decrease.

As the number of individuals in the aging species diminishes, (as can be seen in top right lattice of Figure 1), random variation becomes more important for the aging species, allowing its average fitness to oscillate faster. This can allow for a temporary recovery, as the fitness increases, but it also creates a larger chance for a deleterious change. If that was the whole story, mutation might be able to buy the aging species some time, as it does in the case with no change in the environment, but the senescent species would eventually die.

But there is more. As the species start to compete, the pruning introduced by aging has a non trivial effect. It makes the pressure to adapt or perish stronger on the aging species; as its numbers dwindle, those who do survive are are fitter than those of the non-aging species. The reason for that is subtle. Due to mutation and the random selection of individuals, the average fitness of a new generation is a little larger than that of the previous ones. While it is possible for specific individuals to be lucky and survive for a while, on average, each generation is a little better adapted than the previous one.

Average fitness can be decreasing in time due to environment changes, as per example, in the beginning of the run shown in Figure 2. But this affects every agent equally. When compared to the older generations, new generations have an average value of 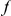 a little higher than the previous one. That way, when senescence kills the elders, it eliminates a slightly worse adapted group. This leaves the space open for the newer, better adapted individuals and it increases their chance to survive. If this improvement is strong enough to compensate for the deaths from age, senescence will be chosen for its own evolutionary merits.

Measurement of the difference between the average fitness of individuals with a difference in age of 4 time steps showed that the system tended to a point where the newer individuals indeed had a fitness that grew up until it was approximately 1% larger. The same increasing pattern in the difference and same difference were observed in all realizations where this difference was measured. Despite this small difference, it was a very consistent difference that was maintained through time and in each realization. As such, it is not the effect of random variation.

Observing the evolution of each realization, we see that the average fitness of the senescent species becomes larger than that of the non senescent one and it stays larger. Fluctuations do happen, since this is a finite system, but the tendency is clear and it was repeated in all runs. Only exceptions where a reversal happened, with the non-aging species showing a higher fitness than the aging one, were due to large fluctuations when one of the two species had very small numbers. Thanks to this tendency to a larger fitness, if the difference in the fitness is large enough, the aging species can slowly drive off the non-aging one.

**Figure 3:**
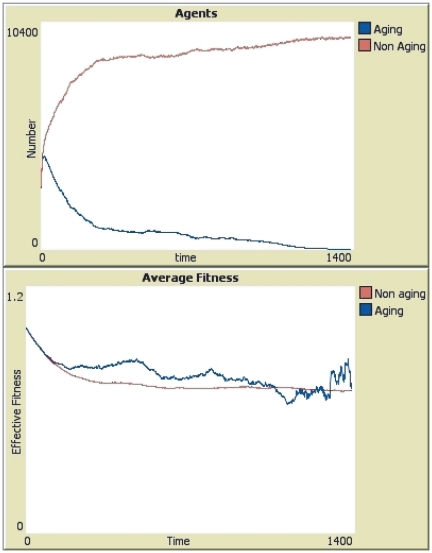
The evolution of a typical run that ended with the defeat of the senescent species. The blue lines correspond to the species where there is senescence (aging) and the red lines to the species where individuals do not age. *Top Panel*: Number of individuals (agents) in each group as a function of the number of generations (time). *Bottom Panel*: Evolution of the average value of the fitness function for each species.

In the cases where the immortal species drive the mortal one to extinction, dynamics is quite similar. A typical run ending with the victory of the non-aging species can be seen in Figure 3. Both cases start in a basically identical way. Once all spaces are occupied and old age starts killing, the aging species start to decline. And, once again, its fitness becomes larger than the fitness of the non aging species. This allows the aging species to postpone extinction but, when a fluctuation in the average fitness make the advantage of the aging species smaller, the senescence starts taking its toll and the aging species is unable to recover from that oscillation.

When some amount of environment change is introduced, obviously, mutation becomes necessary to compensate for that. For a small amount of mutation, as soon as mutation becomes large enough to avoid a crash of the fitness function, the aging species actually shows a tendency to drive the non-aging species to extinction, according to the simulations performed for 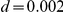, 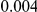, and 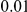. For every value of 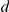, it was observed that, as soon as the mutation 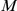 was large enough to prevent a complete collapse of the fitness, it was the aging species that had the advantage of survival. For small changes in the environment (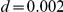), that change was not decisive and a maximum 65% rate of success was observed. That rate declined to zero as 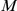 got larger. For 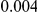, if mutation was just large enough to prevent the collapse, all 20 runs ended with the extinction of the non-aging species. The same effect was observed for faster changes in the environment (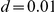).

For all values of the environment change 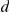, however, as the variation 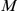 associated with mutation became even larger, the advantage of the non-aging species declined until, for 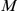 large enough, it disappeared. This meant that too strong variation actually led the aging species to extinction. This is actually to be expected. The survival of the aging species depended on the their average slightly better fitness. With strong variations, this advantage becomes too irregular, with both species able to gain a momentary better fitness.

**Figure 4:**
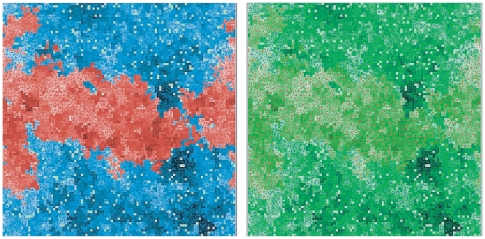
Landscape of fitness values in the middle of a typical one run. These pictures correspond to a moment when the average fitness of the non aging species was 0.89 and that of the aging species, 1.17. *Left Panel* The division between the species is clearly shown, with red representing the non-aging species and blue, the aging species. Both for blue and red, lighter tones indicate a smaller fitness, while darker tones correspond to a higher fitness. *Right Panel* Same circumstances as in the left panel, but only fitness is shown in tones of green, with no distinction between the species, so that their fitness can be better compared.

Figure 4 shows the spatial distribution of fitness in the middle of a typical run, when both species are still competing. The darker colors correspond to a higher fitness. The panel at the right, in green, shows the same moment, but with same green colors for both species, to allow a direct comparison. The right panel figure also shows clearly how the region that is occupied by the blue (aging) species corresponds to a region with average higher fitness than the red region (in that moment, the average fitness for the aging species was 1.17, and for the non-aging one, 0.89). The figures show that, not only the overall average is larger, but this tends to also happens in the frontiers. The larger fitness of the aging species when compared to their actual neighbors of the non-aging species allows its survival.The non-aging species would win, if both values were the same.

#### Exploring the parameter space

We still need to answer how likely agers are to succeed under different conditions in the model, represented by different values of the parameters. To do that, new simulations were prepared in a 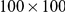 grid and the proportion of times over many realizations that the agers led the non-agers to extinction was registered. As we will see, the extinction of the group of non-agers is a robust result, that can be observed in a wide range of parameter values.

**Figure 5:**
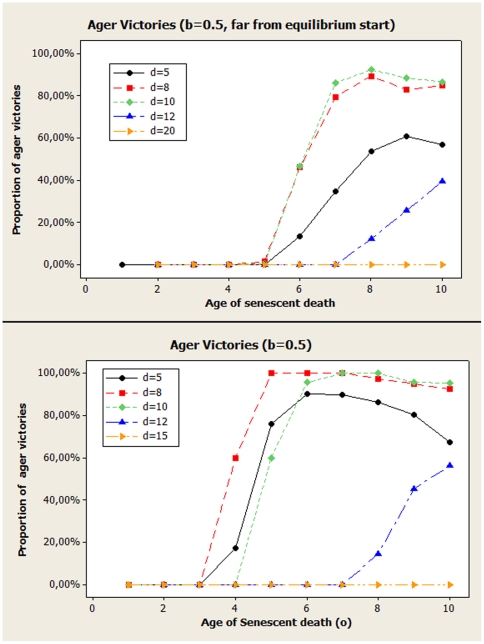
Proportion of simulations that ended with ager victories as a function of the age of death by senescence of the ager population, for slow diffusion (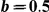). *Top Panel*: Larger initial fitness (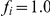), corresponding to a situation that is mostly far of equilibrium. For both simulations, mutation was always 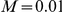 and the the value of 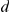 is expressed in multiples of 0.001 for convenience. *Lower Panel*: Lower initial fitness (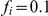), corresponding to a situation that is much closer to equilibrium.

To illustrate the effect of initial fitness, Figure 5 shows results for the proportion of realizations that end in a victory for the ager group, as a function of the age 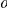 agers die of senescence. Both graphics show the exact same choice of parameters, with the only difference being that for the graphic in the top panel, initial conditions were such that 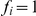, while in the bottom panel, 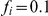, which is closer to most final state average values of 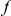 (with the exception of small 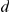, when 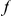 tends to grow). Unless no group has an advantage, for large enough systems, the group that has the advantage should always win. Values different from 0 or 1 (or, possibly, 0.5, if there were really no advantage) represent effects of finite size and noise. It is reasonable to assume that values larger than 50% of ager victories mean the ager group has the advantage and for values lower than 50%, the advantage would be of the non-agers. For values close to 50%, of course,we have either no advantage or a very small one, when noise can interfere with the results more easily and we can't be sure about the winning side.

We see that for starting conditions with larger initial fitness (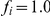), the evaluation is less well defined, with many cases ending with no clear signal about which group is better adapted. This happens because, in most cases, 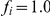 is far from the equilibrium value the 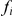 will oscillate around and, as such, there is an important transient that can influence and add more noise to the results. It makes sense to start the system at a more reasonable value and we see that, for 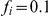, there are a few more well defined answers. The regions where agers have an advantage and were they are at disadvantage are similar as those for 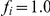, as expected. This shows that, aside of the noise introduced by the initial conditions, the system seems to eventually evolve to a similar result.

Some general features can be observed. As the age of senescent death grows up, very few individuals are likely to reach it and any difference that could exist between both groups becomes negligible. This can be seen as a tendency of every curve to go back to 50% as 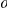 grows up. While agers seem to still have a solid advantage for 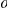 as large as 10, it is visible that all curves start going down. Another obvious feature is that if the agers die too soon (small 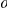), the price of senescence can become too large to be overcome by any fitness advantage. The point where the transition happens is different, but, as soon as 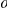 is large enough to overcome that cost, agers start to drive non-agers to extinction for several values of 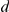.

As discussed before, 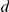 and 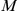 determine the average value of fitness. Larger values of 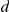 cause more decrease and force 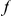 to stabilize at smaller values. For fixed 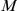, that means the mutation is larger when compared with the fitness and, therefore, a larger 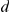 means that mutation is more important. What we see is that when mutation is not so strong (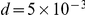) and the agers don't die too soon, they can survive and even get some evolutive advantage, but it is not really clear the advantage is real. By changing 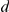 just a small amount, to 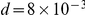, we see that agers can drive non-agers to extinction. However, their advantage disappears again when 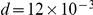. What happens there is that the fitness decreases too much. For 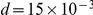, per example, its average is around 0.07, meaning that they are less than 5 time steps away from becoming zero. Also, since 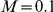, we can conclude that many agents have basically zero fitness. Under those circumstances, it is clear 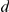 is already unreasonably large.)

**Figure 6:**
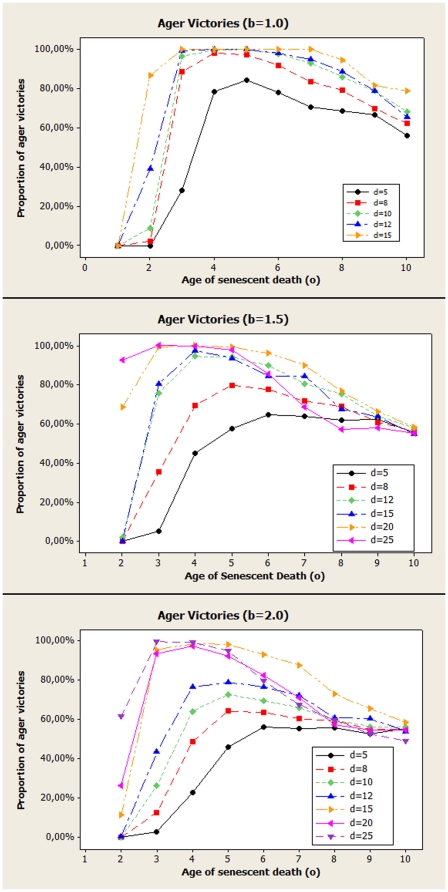
Proportion of simulations that ended with ager victories as a function of the age of death by senescence of the ager population, for different rates of diffusion. All cases started with lower initial fitness (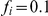). *Top Panel*: Distance of birth 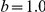. For both simulations, mutation was always 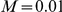 and the the value of 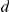 is expressed in multiples of 0.001 for convenience. *Middle Panel*: Distance of birth 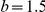. *Lower Panel*: Distance of birth 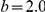.

Figure 6 shows results for smaller initial fitness (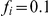) for different values of the dispersal birth distance 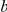, that is, 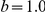, 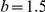, and 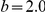. Large values of 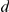 are explored as, due to a increase in competition, it takes a larger 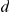 to reach the extreme cases of Figure 5 . The largest possible distance inside a square of size 1 is its diagonal, 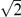, so, for 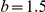, it is already impossible that offspring will compete with its parent. However, the advantage of the ager group is now much clearer, even though direct kin effects should be weaker. Spatial structure still means that the competitors around any agent are very likely to be related to the agent but that chance diminishes as 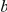 grows. And we can see that agers have an adaptive advantage now that includes a larger range, starting from even smaller ages of senescence.

It becomes clear that, although there is a clear group selection effect in place, the explanation for senescence in the model requires more than that. For the small value of 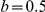, it is very common for an offspring to compete with its parent. Even if this makes the fitness of the agers higher, competition is likely to remain local. Some of the benefits associated with a better fitness can not be obtained, if the higher fitness agers do not spread far before senescence claims its price. As diffusion becomes stronger, competition between groups becomes harder and the adaptative advantages agers have makes it easier for them to invade other areas. Of course, too much mobility can still hurt agers, as it would mean that the benefits of opening spaces to better adapted relatives; when diffusion is too fast it is far more likely a non ager will invade ager territories. A few simulations performed with 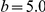 confirm that, as should be expected.

Of course, a number of expected features can also be observed for a stronger diffusion. The tendency that large values of 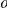 mean none or very small advantage for any group, since almost no individuals die of senescence, is clearly shown, with all curves tending to 50% as 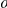 grows. It is also easy to see that there is a minimum value for 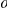, as should be expected, since the curves bend back down as 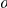 diminishes.

These results present an interesting picture of aging and when it is to be expected. Living beings with limited mobility should have a harder time evolving senescence than those with better mobility. It is to be expected then that animals should present senescence far more often than plants. And, indeed, that is what one observes. Other characteristics not included in this simple model should also be taken into account for real species, of course.

## Discussion

Contrary to the initial guess of Medawar [7], death by senescence has an important evolutionary price for a species that adopt it, as observed [8]. The model presented here when no change is introduced agrees with that result. This clear price meant that senescence was considered for a long time as something that could not be an evolutionary selected characteristic. Here, we have seen otherwise. When there is mutation, agers have a better chance at surviving as they can adapt faster. By introducing gradual change in the environment (the decrease 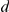 could represent either a change in the external environment or the change due to the competition between the members of both groups, brought by the mutation) , we have seen that extinction of the non-agers is a robust result, observed for a large range of parameter values. Senescence can be chosen by evolutionary dynamics as the best answer to change. Aging produces a pruning effect on the species, eliminating older, slightly less adapted individuals who had managed to survive by chance.

A similar but different idea was proposed by Weismann already in the end of the XIX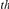 century [43]. He defended that natural selection was the cause of senescence, but he believed the benefits of senescence would be the elimination of individuals who were already somehow damaged by accident and wear. Despite the resemblance and the fact that Weismann was correct about senescence being an adaptation, as shown by many experiments, the model proposed here does not include any damage to the individual. This is a subtle point and deserves a complete explanation, as it might look that way, since the individual fitness does decrease with time. However, this decrease is clearly not one from injuries because the decreased fitness is the one that is transmitted to the new generation. This means the decreased fitness represents the genetic, transmitable material, not damage suffered. It is a true change in outside conditions, either environmental or just a harder competition inside the species and against the other group.

It is true that change in the model is faster than what is observed in the real world. For slow change, that is, small 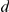, the effect was indeed smaller. Even when change was smaller, per example, when 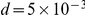, the mutation effect was still strong. The advantage of agers was weaker, but still observed. This means that additional exploration is needed before using the model for a real world situation. But the fact remains that model makes it very clear that aging can really be chosen as an adaptation.

Chance and change were the fundamental keys to this answer. While a larger fitness ensures a better chance of survival, this is only a better probability, both in the model as well as in the real world. If the difference in the fitness between the individuals is not so big, it is to be expected that the better prepared wins only in average. When this is associated with random mutations and environmental changes, an aging species can have an advantage to compensate for the deaths by old age and, in the long run, drive the non-aging species to extinction. A situation based on selection of individuals who live in space, with limited diffusion, can lead to an evolutionary advantage of a senescent species, when change is incorporated. This helps to explain the apparent paradox of why we age. And it illustrates how we still don't understand all consequences of change and random chance in a system as complex as the natural world.
